# Contrasting effects of defaunation on aboveground carbon storage across the global tropics

**DOI:** 10.1038/ncomms11351

**Published:** 2016-04-25

**Authors:** Anand M. Osuri, Jayashree Ratnam, Varun Varma, Patricia Alvarez-Loayza, Johanna Hurtado Astaiza, Matt Bradford, Christine Fletcher, Mireille Ndoundou-Hockemba, Patrick A. Jansen, David Kenfack, Andrew R. Marshall, B. R. Ramesh, Francesco Rovero, Mahesh Sankaran

**Affiliations:** 1 National Centre for Biological Sciences, Tata Institute of Fundamental Research, GKVK Campus, Bellary Road, Bangalore, Karnataka 560 065, India; 2 Nature Conservation Foundation, 3076/5, IV Cross, Gokulam Park, Mysore, Karnataka 570 002, India; 3 Center for Tropical Conservation, Duke University, Durham, North Carolina 27705, USA; 4 La Selva Biological Station, Organization for Tropical Studies, Puerto Viejo de Sarapiquí, Heredia, Costa Rica; 5 CSIRO Land and Water, Tropical Forest Research Centre, PO Box 780, Atherton, Queensland 4883, Australia; 6 Forest Research Institute Malaysia (FRIM), Kepong, Selangor Darul Ehsan 52109, Malaysia; 7 Wildlife Conservation Society (WCS) Congo-Program, Brazzaville B.P. 14537, Republic of Congo; 8 Centre for Tropical Forest Science, Smithsonian Tropical Research Institute, Apartado 0843-03092, Panamá, Republic of Panama; 9 Department of Environmental Sciences, Wageningen University, Box 47, 6700 AA Wageningen, The Netherlands; 10 Department of Botany, CTFS-ForestGEO, NMNH - MRC 166, Smithsonian Institution, PO Box 37012, Washington, District Of Columbia 20013-7012, USA; 11 Environment Department, CIRCLE, University of York, York YO10 5DD, UK; 12 Flamingo Land Ltd., Kirby Misperton, North Yorkshire YO17 6UX, UK; 13 Institut Français de Pondichéry, 11, Saint Louis Street, Pondicherry 605 001, India; 14 MUSE – Science Museum of Trento, Corso del Lavoro e della Scienza 3, 38122 Trento, Italy; 15 School of Biology, University of Leeds, Leeds LS2 9JT, UK

## Abstract

Defaunation is causing declines of large-seeded animal-dispersed trees in tropical forests worldwide, but whether and how these declines will affect carbon storage across this biome is unclear. Here we show, using a pan-tropical data set, that simulated declines of large-seeded animal-dispersed trees have contrasting effects on aboveground carbon stocks across Earth's tropical forests. In our simulations, African, American and South Asian forests, which have high proportions of animal-dispersed species, consistently show carbon losses (2–12%), but Southeast Asian and Australian forests, where there are more abiotically dispersed species, show little to no carbon losses or marginal gains (±1%). These patterns result primarily from changes in wood volume, and are underlain by consistent relationships in our empirical data (∼2,100 species), wherein, large-seeded animal-dispersed species are larger as adults than small-seeded animal-dispersed species, but are smaller than abiotically dispersed species. Thus, floristic differences and distinct dispersal mode–seed size–adult size combinations can drive contrasting regional responses to defaunation.

## Abstract (web summary)

Defaunation is linked to the decline of tree species that depend on large animals for seed dispersal, but it is unclear if this affects carbon storage. Here the authors show that defaunation effects on carbon storage vary across continents, driven by relationships between seed dispersal strategies and adult tree size.

## Graphical abstract

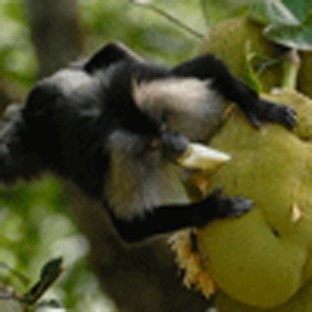

Anthropocene defaunation is amongst the most pervasive drivers of Earth's ongoing biodiversity crisis^1^,^2^,^3^,^4^. The rates at which the tropics are losing vertebrate species are amongst the highest globally, with larger species being particularly vulnerable to population declines and extirpations^1^,^5^,^6^,^7^. Declines of large vertebrates can have widespread, cascading effects on community- and ecosystem-level processes, because smaller organisms are often unable to perform the ecological roles of larger vertebrates^1^,^2^. In tropical forests, where the majority of tree species depend on vertebrate frugivores for seed dispersal^8^, theory and empirical evidence indicate that defaunation can substantially alter the composition of tree communities, through effects on seed dispersal processes^9^,^10^,^11^ and cause tree recruitment to shift towards smaller-seeded animal-dispersed and abiotically dispersed species^12^,^13^,^14^,^15^. Declines of large vertebrate frugivores can result in reductions of up to 60% in the abundance of tree species that depend on them for seed dispersal^16^, with resultant declines of over 25% in average seed sizes of adult tree communities^13^. With tropical forests representing one of the largest terrestrial carbon pools and playing a critical role in regulating global climate^17^,^18^, understanding whether and how such defaunation-driven declines in large-seeded species can affect carbon storage in tropical forests across the globe is critical^19^,^20^.

The consequences of defaunation-driven shifts in tree community composition for carbon stocks will depend on how small-seeded and abiotically dispersed species differ from large-seeded animal-dispersed species in their potential to store carbon^19^. Recent work from Amazonian and Atlantic forests suggest that defaunation has the potential to drive aboveground carbon losses through shifts towards tree communities with less-dense wood because small-seeded animal-dispersed species in Neotropical forests tend to have lower wood densities than larger-seeded species^19^,^20^. However, whether defaunation has similar effects on the carbon storage potential of other tropical forest communities that have different floristic compositions, and the potential mechanisms underlying such effects at the global scale, remain unknown.

Here, we present a pan-tropical assessment of the potential effects of defaunation on aboveground carbon storage by simulating extirpations of large-seeded animal-dispersed species from 10 relatively undisturbed tropical forest tree communities spanning four continents. These sites span a broad range of floristic types and form a gradient in the prevalence of animal-mediated seed dispersal from highest in tropical Africa, India and the Americas, to lowest in the more wind-dispersed assemblages of Australia and Southeast Asia. Our data set includes stem measurements and functional traits of over 25,000 trees of ∼2,500 species representing at least 785 genera and 155 families.

We examined the potential effects of defaunation on aboveground carbon storage by simulating declines in relative abundances of large-seeded animal-dispersed species within tree communities at each site. In our simulations, where we held the total basal area of each site constant, we removed individuals of large-seeded animal-dispersed species and replaced the lost basal area through a random draw of individuals from the remaining pool of individuals within each community^21^ (Methods section). We simulated four levels of defaunation-driven losses of large-seeded animal-dispersed species by removing 25, 50, 75 and 100% of these individuals from each of our sites. This, on average, reduced community-weighted seed size of animal-dispersed species across sites by 6, 14, 23 and 33%, respectively. Aboveground carbon stocks of the original and simulated communities were then estimated using data on tree diameters and species wood density in a general biomass equation for tropical forest trees^22^.

In addition to the above simulations, we also simulated two control scenarios. First, because natural tree communities typically contain far greater numbers of smaller trees than larger individuals, removal of large individuals followed by replacement using random draw from the remaining pool to make up original basal areas, as in our simulations, can reduce carbon stocks simply as a result of larger individuals being replaced by multiple smaller individuals. To distinguish the effects of losing individuals of large-seeded species from such random ‘sampling' effects, we also simulated a set of ‘individual-based' control scenarios in which equal numbers of individuals as those lost from defaunation scenarios were removed at random from original tree communities. The lost basal areas were replaced through random draw of individuals from the remaining pool, as was done in the defaunation scenarios. Second, removing individuals can sometimes lead to incidental species extinctions and reduce overall species richness, which in turn, can reduce the carbon storage potential of forest stands^21^. To distinguish the effects of defaunation-driven losses of large-seeded species from those of random species loss *per se*, we also simulated a second set of ‘species-based' control scenarios in which equal numbers of species as those lost in the defaunation scenarios were removed based on random selection from the overall pool of species, and lost basal areas replaced through random selection of individuals from the remaining pool (Methods section).

Our simulations of declines in large-seeded species in tropical forests across four continents suggest that (i) defaunation effects on aboveground carbon storage are likely to differ between regions, contingent on floristic composition. Carbon losses are expected in forests of the Americas, Africa and South Asia where tree communities are dominated by animal-dispersed species, but not in forests of Southeast Asia and Australia where large, abiotically dispersed species are more prevalent; (ii) at the pan-tropical scale, changes in aboveground carbon storage are driven by changes in stand volume rather than wood density; (iii) shifts in carbon storage following defaunation are underlain by globally consistent relationships between species' dispersal mode, seed size and potential adult size, wherein large-seeded animal-dispersed species attain larger adult sizes and therefore have more volume as adults than small-seeded animal-dispersed species, but are smaller than abiotically dispersed species. As a growing proportion of tropical forests are facing defauntion due to hunting, fragmentation, logging and other anthropogenic disturbances, conserving large tree species and their seed dispersers will be important for climate regulation.

## Results

### Defaunation scenarios and aboveground carbon storage

**Figure 1:**
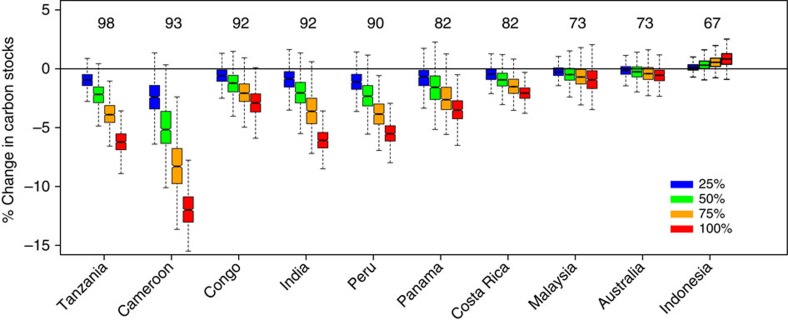
Effects of simulated removal of large-seeded animal-dispersed species on aboveground carbon storage. Box-and-whisker plots depicting changes relative to original carbon stocks for 1,000 simulation runs following removal of 25, 50, 75 and 100% of individuals belonging to large-seeded animal-dispersed tree species. Numbers above boxes represent the relative abundances (%) of animal-dispersed species at each site.

Simulated losses of large-seeded, animal-dispersed tree species decreased estimated aboveground carbon stocks by up to 5% under the 50% removal scenario (half of the individuals of large-seeded species lost from communities), and by as much as 12% under the 100% removal scenario, that is, complete extirpation of all large-seeded animal-dispersed species in communities (Fig. 1, Supplementary Tables 1 and 2). In contrast, carbon storage was largely unaffected in the individual- and species-based control scenarios, with shifts in carbon stocks ranging between −0.4% and +0.1% at the highest extirpation levels across the 10 sites (Supplementary Fig. 1, Supplementary Tables 1 and 2), suggesting that changes in carbon stocks in the defaunation scenarios were indeed primarily driven by reductions of large-seeded animal-dispersed tree species. Overall, simulated losses of large-seeded species reduced carbon stocks in the African, Indian and American tree communities, which had relatively high abundances (>80% of all individuals) of animal-dispersed species (Fig. 1 and Supplementary Table 1). Aboveground carbon stocks in these communities decreased by 1–5% under the 50% removal scenario, and by 2–12% under the 100% extirpation scenario (Fig. 1 and Supplementary Table 1). In Australia and Southeast Asia, where abundances of animal-dispersed tree species are lower (<75% of all individuals), carbon stocks either decreased or increased marginally (Fig. 1 and Supplementary Table 1). These results are in accordance with the limited empirical evidence that currently exists, which shows carbon losses associated with defaunation in an African forest^23^ but not in a Southeast Asian site^15^. These patterns remained unchanged when we used fixed, rather than site-specific cutoffs (75th percentile; Methods section) to classify large-seeded species for our simulations (Supplementary Fig. 2).

### Wood density and woody volume

**Figure 2:**
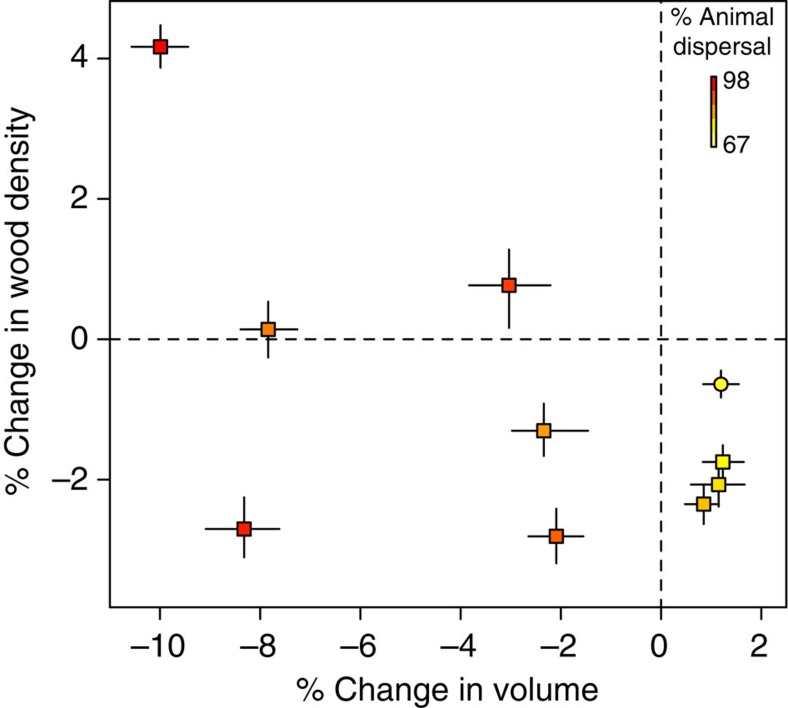
Changes in stand volume and wood density following simulated extirpations of large-seeded animal-dispersed species. Points represent medians of percentage change in stand volumes and community-weighted wood densities in response to simulated extirpation of all large-seeded animal-dispersed species. Corresponding inter-quantile ranges of change in volume and wood density across 1,000 simulation runs at each site are depicted using lines. Sites that show decreases and increases in carbon stocks are depicted with squares and circles, respectively. The colour gradient depicts variation in the relative abundances of animal-dispersed tree species ranging from yellow (low) to red (high).

Theoretically, for a given basal area, changes in aboveground carbon storage can result from shifts in either wood density or stand volume, or both^24^. We related changes in carbon stocks to overall changes in stand wood densities and volumes by assessing the independent responses of their respective terms within the allometric equation used for biomass estimation (Methods section). Because our plot data sets only contain measurements of tree diameters and not heights, we estimated tree heights from diameters using a model derived from a large pan-tropical data set of tree measurements, with height increasing monotonically with diameter^22^. In our simulations, changes in carbon stocks following the loss of large-seeded species were strongly correlated to shifts in stand volumes (Pearson correlation *R*_p_=0.84, *P*<0.01), but not stand-level wood densities (*R*_p_=−0.04, *P*>0.90). Stand volumes decreased at sites dominated by animal-dispersed species (for example, Africa) while they increased at sites with relatively higher abundances of abiotically dispersed species (for example, Southeast Asia) (Fig. 2, Supplementary Fig. 3a, Supplementary Table 1). In contrast, wood density showed no consistent response at sites dominated by animal-dispersed species, but decreased at sites with relatively higher abundances of abiotically dispersed species (Fig. 2, Supplementary Fig. 3b, Supplementary Table 1).

### Seed dispersal-woody trait relationships

**Figure 3:**
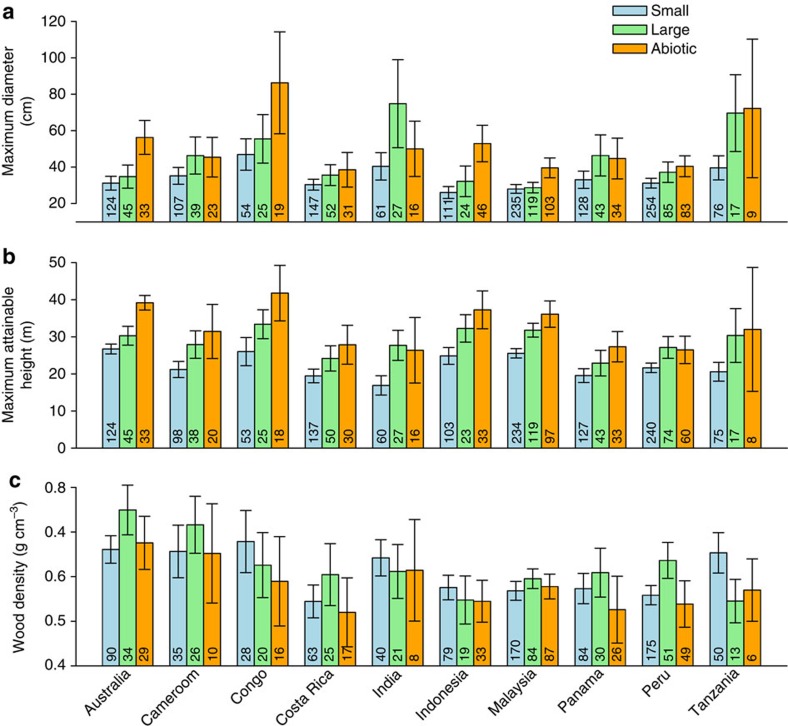
Relationships of potential adult size and wood density with seed size and dispersal mode. Large-seeded animal- and abiotically dispersed tree species have greater maximum diameters (**a**) and maximum attainable heights (**b**) than small-seeded animal-dispersed species, consistently across sites. (**c**) Wood densities do not consistently differ with seed sizes of animal-dispersed species and are lowest among abiotically dispersed species (Supplementary Table 3). Bars represent means and error bars 95% confidence intervals. The numbers of species belonging to each category are reported within bars. Species' maximum diameters are obtained from the plot data set while maximum attainable height and wood density are collated from secondary sources (Methods section).

When basal area is constant, changes in stand volumes can result from shifts in tree size class distributions, because individuals with larger basal diameters, and correspondingly greater heights, contain more volume than an equal basal area made up of smaller individuals (Supplementary Fig. 4). In our simulations, losses of large-seeded species were accompanied by reductions in relative abundances of large trees (trees of basal diameter ≥70 cm (ref. 25)), and thus stand volumes, in communities dominated by animal-dispersed species, but not in communities with more abiotically dispersed species, or in the control scenarios (Supplementary Fig. 3a,c, Supplementary Table 1). These patterns are explained by consistent empirical relationships between species' seed dispersal traits and potential adult size, as indexed by maximum diameter and maximum attainable height, across ∼2,100 of the 2,500 species in our data set (Fig. 3a,b). Large-seeded animal-dispersed species in our plots had 24% greater maximum diameters on average than did small-seeded animal-dispersed species, but were 14% smaller on average than abiotically dispersed species across sites (Fig. 3a, Supplementary Table 3). We also found a very similar relationship between seed dispersal traits and maximum attainable height, collated from secondary sources (Methods section), wherein maximum attainable heights were 26% greater for large-seeded animal-dispersed species than for small-seeded animal-dispersed species, but 12% lower than for abiotically dispersed species (Fig. 3b, Supplementary Table 3). Extirpations of large-seeded species, particularly where abiotically dispersed species are rare, can therefore reduce the representation of species that attain relatively large adult sizes, and thus drive declines in stand volume and carbon stocks in these communities^26^. In regions where large, abiotically dispersed species are more abundant, such as the Dipterocarp forests of Southeast Asia^27^, increased abundances of these species following losses of large-seeded, animal-dispersed species can potentially maintain or even increase stand volumes and carbon storage^15^.

In contrast to potential adult size, wood density showed little variation across seed sizes of animal-dispersed species and was lowest among abiotically dispersed species in our data set (Fig. 3c, Supplementary Table 3). At a pan-tropical scale, extirpations of large-seeded animal-dispersed species are therefore unlikely to consistently reduce stand wood densities (Fig. 2, Supplementary Table 1). Thus, although declines in large-seeded animal-dispersed species may reduce carbon stocks by promoting species with lower wood densities in some tropical forests^20^,^28^, the importance of wood density as a factor driving defaunation effects on carbon storage across the tropical forest biome appears to be limited^29^.

## Discussion

**Figure 4:**
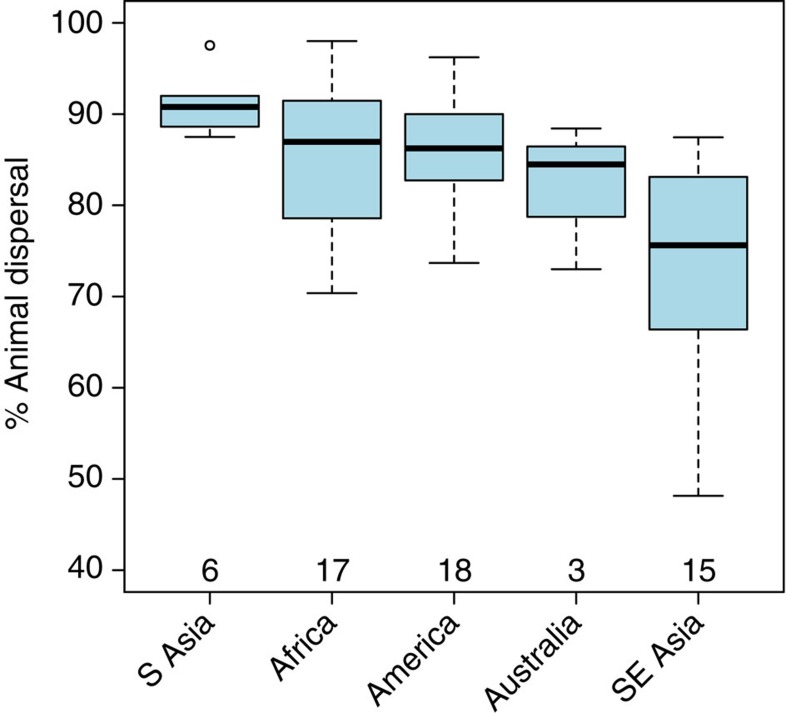
Variation in relative abundances of animal-dispersed species across major tropical forest regions. The box-and-whisker plots show that tropical forest tree communities in most regions have high-relative abundances of animal-dispersed species, with the exception of Southeast Asia where some communities comprise over 50% abiotically dispersed species. Numbers below boxes represent the number of sites from which data were obtained for each continent (see Supplementary Fig. 5 and Supplementary References 1–44 for more details).

Presently, an estimated 88% of all tropical forests face the threat of defaunation through the combined effects of hunting, habitat fragmentation, selective logging and other forms of anthropogenic disturbance^3^, with animals that disperse large-seeded tree species amongst the most vulnerable^30^. Our results suggest that declines of these large-bodied dispersers can have contrasting effects on aboveground carbon storage across tropical forests, contingent on local species pools, relative abundances of animal-dispersed species in communities and the relationships between dispersal mode, seed size and adult size. Given that tropical forests of the Americas, Africa and South Asia, which together account for over 75% of all forests within the biome^31^, are primarily composed of animal-dispersed tree species (Fig. 4, Supplementary Fig. 5), our results suggest that defaunation could have marked negative effects on aboveground carbon storage in tropical forests overall. Although accurate extrapolation of our results to all tropical forests is not possible at this time, a rough estimate of the overall magnitude of likely carbon losses due to defaunation does provide some perspective. If tropical America and Africa lose 2.1% of their 124 Pg aboveground carbon stocks^32^, as predicted on average by our 50% extirpation scenarios (Supplementary Table 1), this would release ∼2.6 Pg of carbon, or 14 years' worth of Amazonian deforestation^33^, to the atmosphere. Such losses can be further exacerbated when defaunation additionally favours other carbon-poor woody life forms such as lianas and palms^29^,^34^,^35^.

**Figure 5:**
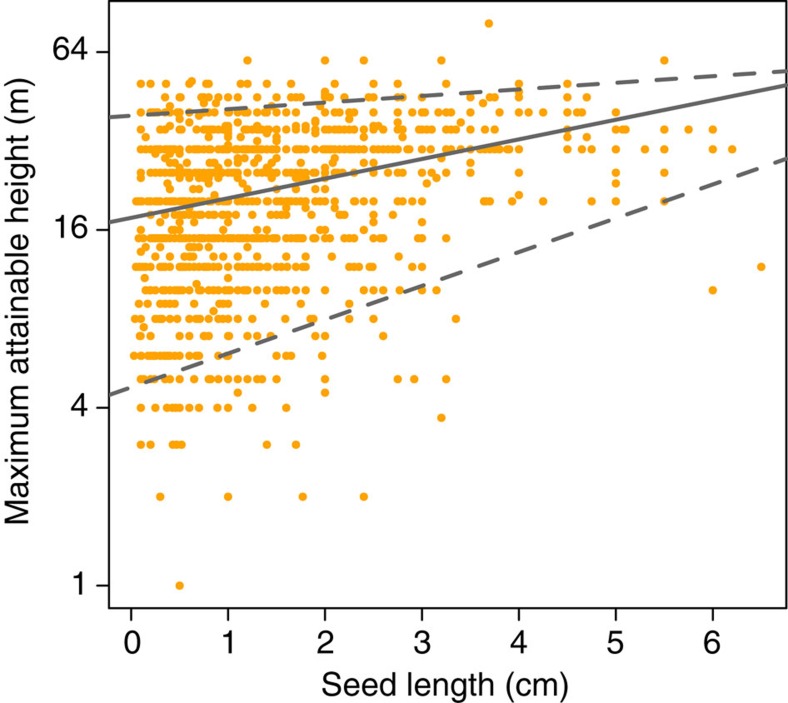
Seed size-maximum attainable height relationship. The relationship of maximum attainable height (log scale) with seed length for animal-dispersed tree species across all 10 study sites, with fitted lines representing median (solid line) and 95 and 5% regression quantiles (dashed lines). The wedge shape of this point cloud is characteristic for seed size maximum-attainable height relationships^36^.

Ultimately, the magnitude of aboveground carbon losses will depend on the identity of tree species that replace the individuals lost in defaunated forests. For example, although small-seeded species are considerably shorter on average as adults than large-seeded ones (Fig. 3b), the tallest species of both groups have similar heights, resulting in the characteristic wedge shape of seed size-maximum height relationships (Fig. 5) (ref. 36). While our estimates are based on all trees having an equal probability of replacing large-seeded species, carbon losses could be mitigated if the taller among small-seeded species were to disproportionately benefit in defaunated forests. However, our understanding of such processes is currently limited by a paucity of empirical data on forest dynamics in defaunated forests. Changes in aboveground species compositions are also likely to affect carbon cycling and storage in leaf litter and soils^37^,^38^, but the effects of defaunation on these important components of the tropical forest carbon cycle are virtually unknown. Likewise, the time frame over which such changes may occur is unknown at this time, but is likely to be much longer than more immediate drivers of carbon losses such as deforestation or logging. More long-term empirical data on tree community dynamics and carbon budgets of defaunated forests, combined with temporally explicit models^39^, are urgently needed to better predict magnitudes of these changes and the time frames over which they may occur. Our results highlight that defaunation can have contrasting effects on carbon storage across the global tropics, contingent on the local species compositions of tree communities. Whereas previous discussions have focused on shifts in wood densities in some regions^20^,^28^, we illustrate that declines of large tree species and concomitant reductions in stand volumes may be the primary mode of defaunation-driven carbon losses in tropical forests at the global scale. Given the disproportionate contributions of large trees to aboveground carbon stocks^25^, conserving their seed dispersers across the tropics is likely to be important for climate regulation.

## Methods

### Vegetation plot data

Tree community data consisting of measurements of all trees ≥10 cm diameter at breast height (DBH) were obtained from 58 vegetation plots with a total sampled area of 55 ha from 10 sites spanning the humid tropics (Supplementary Table 4). Data were obtained from three sources: (a) 1 ha plot data available from the Tropical Ecology Assessment & Monitoring Network (TEAM) open data portal (http://www.teamnetwork.org/), (b) 1 ha plot data from an open access data set published by Ramesh *et al*.^40^ and (c) 0.5 ha plot data from an open access data set published by Bradford *et al*.^41^. For multi-temporal data sets containing repeated measures, only data from single surveys conducted during 2011–2013 were considered. Species names were checked and standardized in accordance with currently accepted taxonomies^42^. Tree DBH values were cross-checked against measurements from previous survey periods, when available, to detect and correct obvious errors. Entries for conifers (0.03% of all individuals recorded in plots) and woody vegetation other than trees, such as lianas (0.09%) and palms (5%), were excluded because the general allometric equations used for biomass estimation (see below) are not applicable to these groups. However, in the case of palms which are quite common in some tropical forests, we performed a comparison between simulations run with palms excluded and simulations with palms included, using the same biomass equation for palms as for trees^43^ and found that excluding palms had little influence on overall carbon responses (Supplementary Fig. 6). The data set comprised 26,512 trees, including 23,822 trees identified to the species level and 1,660 identified only to the genus level. The data set also comprised 1,030 trees that were not identified to the genus level, which were excluded from subsequent analyses.

### Functional traits data

Data were collected on seed dispersal mode, seed length, wood density and maximum attainable height for the species in the vegetation plot data sets (key secondary data sources listed in Supplementary References 45–77). First, species were classified based on seed dispersal mode into four categories: (a) animal (vertebrates and invertebrates), (b) wind, (c) unassisted (explosive dehiscence, gravity and water) and (d) multiple (modes combining a with b and c). Groups a and d were then aggregated under the category of animal-dispersed species while groups b and c were grouped as abiotically dispersed species. Data on seed dispersal modes were primarily obtained from the Seed Information Database of Kew Gardens^44^, as well as floras and scientific studies focusing on seed dispersal in tropical forests. In the absence of species-level classifications, dispersal mode was assigned based on data from congeners or using resources which provide genus-level classifications^45^,^46^.

Species' seed sizes were estimated as the average length (cm) of the longest seed axis. This metric was selected to index seed sizes in this study because (a) it is the most widely reported measure of seed size, and (b) it shares generally consistent relationships with dimensions of other seed axes^13^,^47^. Comparison of 295 tropical tree species in our database showed that seed lengths were closely correlated with seed dry weights (Spearman correlation *R*_s_=0.87, *P*<0.0001), another trait widely used to index seed size in studies of animal seed dispersal. Seed lengths were obtained from direct measurements, as well as from regional floras accessed through online databases, the Biodiversity Heritage Library (http://www.biodiversitylibrary.org/), journal articles and digital images of specimens. In case of images, seed lengths were estimated using the reference scales provided. Large-seeded animal-dispersed species were classified as those larger than the 75th percentile seed length amongst all animal-dispersed species within each community (Supplementary Data 1). This approach to defining large-seeded species was used because although size distributions of both seeds and their dispersers are known to vary considerably across tropical regions^46^, guideline values for defining large-seeded species are not available for all sites. However, where such values were available from other sites in the Americas (1.5 cm) (refs 48, 49), Africa (1.8 cm) (ref. 13) and the Orient (1.5–2.0 cm; refs 15, 50), we found them to be similar to the 75th percentile cutoffs used in this study. When species-level seed lengths were not available for defining large-seeded species, proxies derived from genus-level information were used to classify species for the simulations. These values were obtained from the following sources in decreasing order of preference:

1. Seed length as a function of fruit length: data on fruit lengths are generally reported alongside seed lengths in most flora resources, and are often available even when seed lengths are not reported. We developed genus-wise linear models of seed length as a function of fruit length, and observed a strong fit amongst models (mean *R* ^2^=0.92) based on an assessment of 982 species across 139 genera. Thereafter, in genera that had at least three species contributing to the model, these models were used to predict seed lengths for congeners which only had data on fruit lengths.
2. Genus-level seed length: genus-level seed lengths were obtained from Dennis *et al*. 45. Based on a comparison of 1,125 species across 325 genera, we observed that seed lengths at the species and genus levels are strongly positively correlated (*R*_p_=0.69, *P*<0.0001).

Wood density (g cm^−3^) data were obtained from primary sources and from the Global Wood Density Database^51^,^52^. When using secondary sources, only data that were collected in the same continent as the location of the target species were used, that is, for example, only records from Africa contributed to wood density estimates for African species, and so on. In cases where species-level wood densities were not available, average values across members of the same genus or family were assigned. These genus- and family-level estimates were used only in the simulations, and not for assessing relationships with other species traits.

Maximum attainable heights (*m*) were obtained from regional floras accessed through online databases and the Biodiversity Heritage Library (http://www.biodiversitylibrary.org/). Among species for which data on maximum height and maximum diameter were available from floras, there was a strong and positive correlation between these two indices of adult size (*R*_s_=0.75, *P*<0.0001, *N*=813).

### Carbon storage estimation

Carbon stored by individual trees was estimated using the following equation developed by Chave *et al*.^22^ for moist forests:


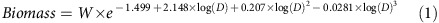



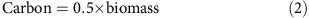


where *W* is wood density in g cm^−3^, and *D* is DBH in cm.

This equation was selected because (a) it is developed based on a large pan-tropical data set of tree measurements, and (b) height measurements are not required for carbon estimation (but height-diameter relationships are implicit), which is crucial because the plot data sets used here did not contain information on tree heights. Moreover, the structure of equation 1 permits ready deconstruction into its wood density (*W*, term 1) and volume (term 2 comprising the DBH-related terms within the exponent) components. Stand-level carbon stocks and volume estimates were obtained by summing over all individuals within each site, and dividing by the total sampled area for per-hectare estimates. Stand-level wood density was estimated as the average wood density across species, weighted by basal area^25^.

Although the allometric equation used here is known to overestimate aboveground biomass^53^, we make no between-site comparisons of aboveground biomass, and thus, do not expect our choice of equation to bias our results in any way.

### Simulations

For the simulations, data from plots within sites were pooled to create single community data sets per site, ranging in sampled area from 3 to 6 ha (Supplementary Table 4). Shifts in tree community composition and aboveground carbon storage were simulated using a two-step procedure, comprising a removal, followed by a recovery step. In the removal step, a certain number of individuals (*N*_Rem_) were removed from tree communities following a set of rules (see below), which resulted in a reduction of total basal area (BA_Loss_), as well as incidental reductions in species richness (*S*_Loss_). In the recovery step, BA_Loss_ was recovered (with a target accuracy of ±1%) by repopulating the community with individuals (N_Rec_) selected through a random draw, with replacement, from the remaining pool of individuals^21^. As the target was to recover lost basal area, and not tree densities, *N*_Rem_ and *N*_Rec_ could differ in value.

Three sets of scenarios were simulated for each community, namely (1) a defaunation scenario in which declines of large-seeded animal-dispersed species were simulated, and two control scenarios to distinguish defaunation effects from (2) simulation artefacts resulting from removing and replacing individuals, and (3) the effects of species loss *per se*.

In the defaunation scenario, declines of species dispersed by large animals were simulated by randomly removing individuals belonging to large-seeded animal-dispersed (>75th percentile seed length) tree species and repopulating the community with individuals drawn at random from the remaining pool, to recover original total basal areas. Four levels of defaunation-driven losses were simulated, with *N*_Rem_ equalling 25, 50, 75 and 100% of individuals belonging to large-seeded animal-dispersed species, respectively. One thousand iterations were simulated for each of the four levels at each site.

The first control scenario was simulated to distinguish the effects of defaunation on aboveground carbon stocks from numerical effects arising solely from the removal and replacement of individuals in the simulations. One control scenario was simulated for every iteration of the defaunation scenario, with *N*_Rem_ values matching those of the corresponding defaunation scenario, but with removal of individuals through a random draw from the overall pool, irrespective of seed dispersal category. Recovery of lost basal areas was simulated through random draws of individuals from the remaining community.

The second control scenario was simulated to control for the effects of species loss *per se*, which could accompany the removal of individuals in the defaunation scenarios, and is bound to occur in the 100% removal scenario. One control scenario was simulated for every iteration of the defaunation scenario, with *S*_Loss_ values matching those of corresponding defaunation scenarios, but with removal of species through a random draw from the overall pool. Recovery of lost basal area was simulated through random draw of individuals from the remaining community.

The R code used to run the simulations has been uploaded to a GitHub repository and is available here: https://github.com/aosuri/defaunation_carbon_project.

### Analysis

Before analysis, each response variable was recalculated as the percentage change from the corresponding value for the original community. The distributions of percentage change in carbon stocks, stand volumes, basal area-weighted wood densities and relative abundances of large trees (≥70 cm DBH, following Slik *et al*.^25^) in the defaunation and control scenario simulations are graphically represented using boxplots. We considered cases where the entire inter-quantile range of a response in any given scenario does not overlap with zero to indicate consistent effects of species/individual extirpations. The percentage of simulation runs in each scenario that showed declines in aboveground carbon stocks was also recorded. Defaunation effects were estimated as the difference in median percentage change between the defaunation and control scenarios for each response variable. We present these effect sizes without conducting statistical significance tests to estimate *P* values, as recommended by recent papers on the inappropriateness of significance testing in simulation studies^54^,^55^.

The R statistical environment version 3.0.2 (ref. 56) was used for running simulations, analyses and preparation of figures.

## Additional information

**How to cite this article:** Osuri, A. M. *et al*. Contrasting effects of defaunation on aboveground carbon storage across the global tropics. *Nat. Commun.* 7:11351 doi: 10.1038/ncomms11351 (2016).

## Supplementary Material

Supplementary Information Supplementary Figures 1-6, Supplementary Tables 1-4 and Supplementary References

Supplementary Data 1 Species details table, including data on site, family, species name in database, accepted species name (according to http://www.theplantlist.org/) and seed dispersal category assigned. Seed dispersal categories include large-seeded animal-dispersed (L), small-seeded animaldispersed (S) and abiotically-dispersed (A)
