# Supplementary material for: Contrasting effects of defaunation on aboveground carbon storage across the global tropics
Source: Nat Commun. 2016 Apr 25;7:11351. doi: 10.1038/ncomms11351 (PMC4848488; doi:10.1038/ncomms11351)
Supplement: Supplementary Information — Supplementary Figures 1-6, Supplementary Tables 1-4 and Supplementary References [file ncomms11351-s1.pdf]

Supplementary Figures

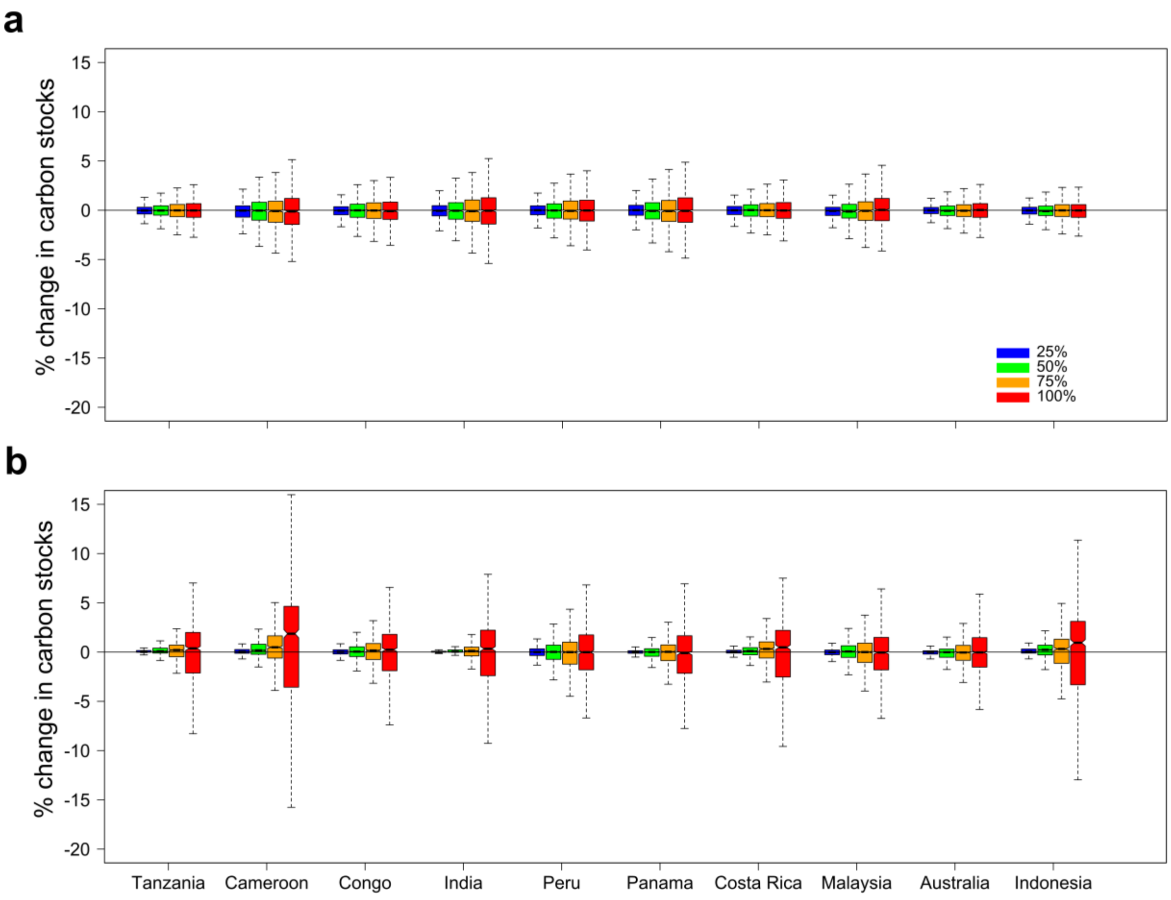

Supplementary Figure 1. Changes in carbon stocks in two control scenarios in which equal numbers of (**a**) individuals and (**b**) species as those extirpated in the 25%, 50%, 75% and 100% levels of the defaunation scenarios (manuscript Fig. 1) were removed

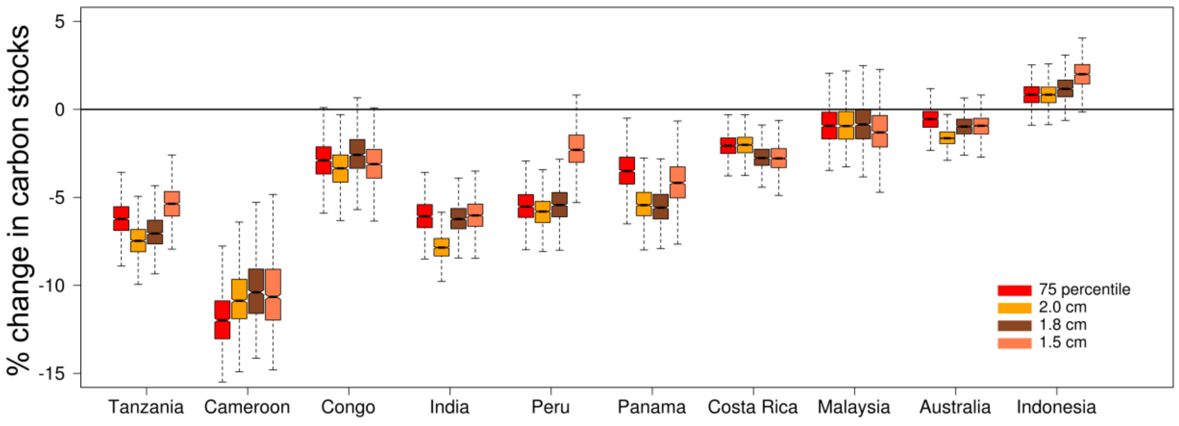

Supplementary Figure 2. Sensitivity of carbon storage responses to the threshold seed size used to define large-seeded species. Boxes represent change in carbon stocks for 100% removal of large-seeded animal-dispersed species using different seed length thresholds to define large-seeded species, namely, the 75th percentile seed length for each community, 2.0 cm, 1.8 cm and 1.5 cm. Simulations using different definitions consistently produced similar results

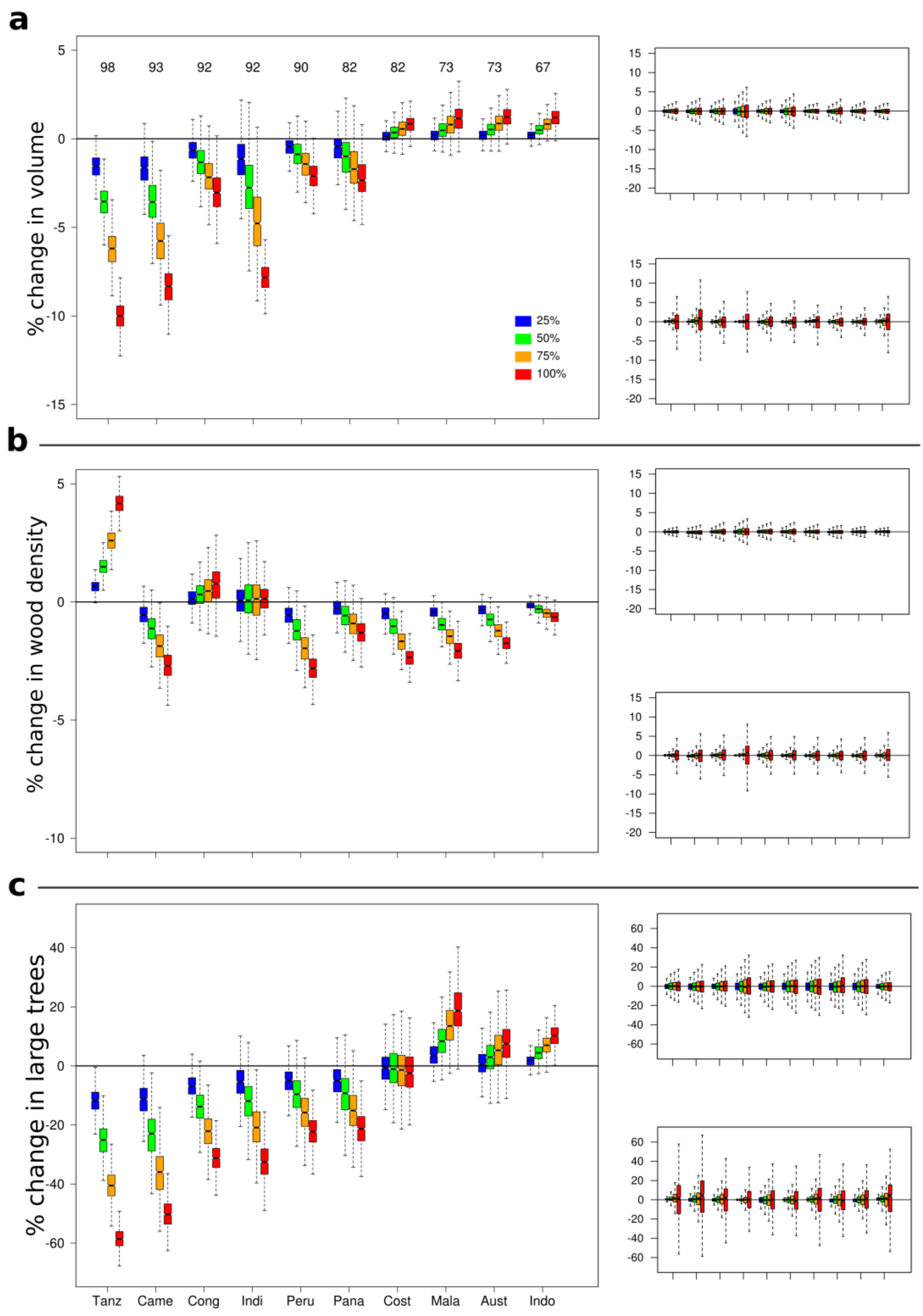

Supplementary Figure 3. Changes in (a) stand volumes, (b) community-weighted wood densities and (c) relative abundance of large trees (≥70 cm DBH) relative to original values for 1000 simulation runs following removal of 25%, 50%, 75% and 100% of individuals belonging to large-seeded animal-dispersed tree species. The small plots towards the right of each panel represent corresponding responses in control scenarios in which equal numbers of individuals (above) and species (below) as those extirpated in the defaunation scenarios were removed

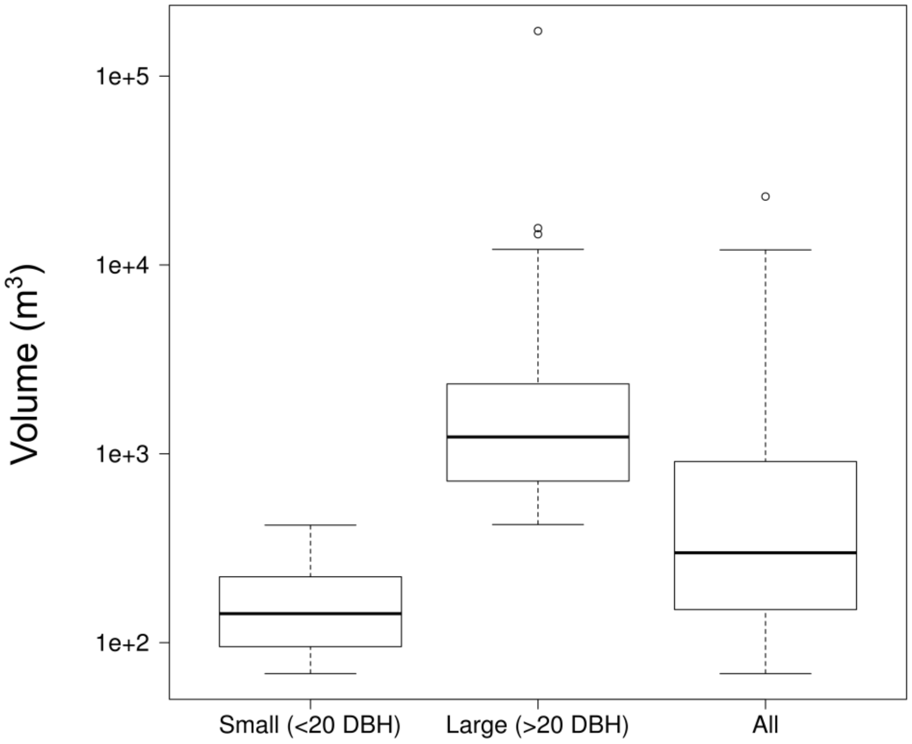

Supplementary Figure 4. Boxplots showing that for a given basal area ($30m^2$ ha^{-1}), forest stands comprising only small trees (<20 cm DBH) have less volume than stands comprising large trees (>20 cm DBH) or mixed stands with small and large trees (All). Equal basal area stands were assembled through simulations by randomly drawing individuals from the pool of small trees, large trees and all trees within the overall pool

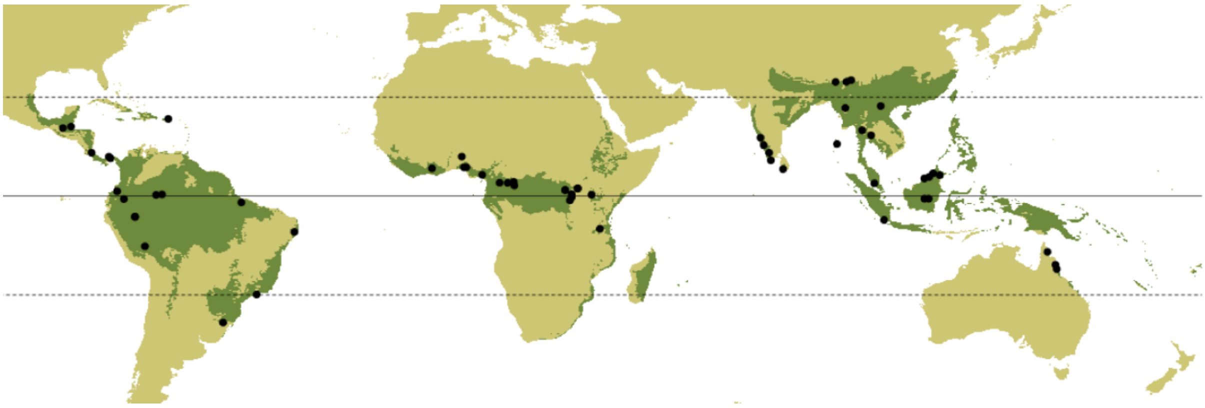

Supplementary Figure 5. Map showing the locations of 59 tree community datasets that were used to assess the prevalence of animal-dispersed tree species across tropical forest regions (also see Fig. 4 of the main text and Supplementary References 1-44). Regions shaded green depict "Tropical and subtropical moist broadleaf forests" as defined in WWF's Terrestrial Ecoregions of the World[78], downloaded from http://www.worldwildlife.org/publications/terrestrial-ecoregions-of-the-world

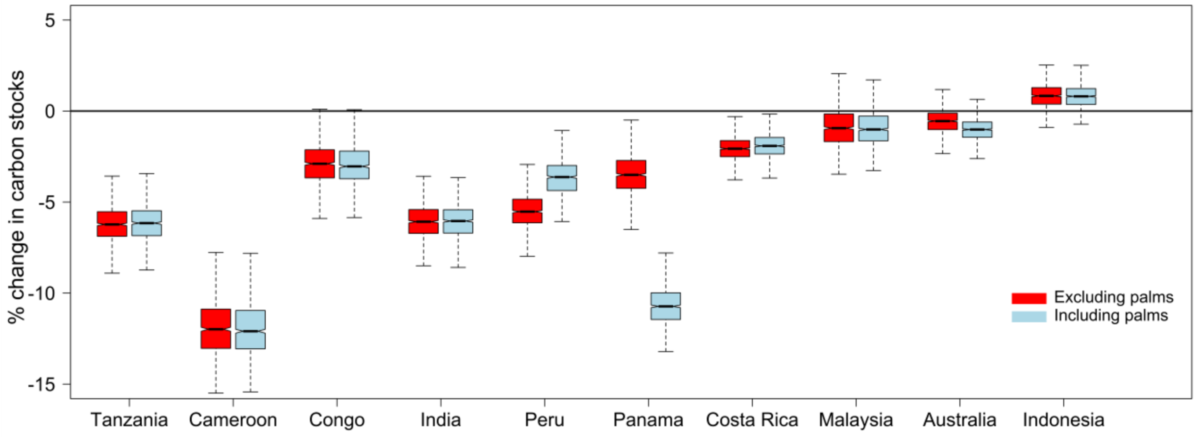

Supplementary Figure 6. Sensitivity of carbon storage responses to exclusion of palms from the simulations. Boxes represent simulated changes in carbon stocks following removal of all large-seeded animal-dispersed species from tree communities with palms either excluded (red) or included (blue). Simulations excluding and including palms consistently produced similar results

Supplementary Tables

Supplementary Table 1: Simulated defaunation effects on carbon stocks, volume, wood density and relative abundances of large trees. Differences in median % change between defaunation and individual-based control scenarios (D-C1) and defaunation and species-based control scenarios (D-C2) for these responses at different extirpation levels are presented

Response	Country	25% removal		50% removal		75% removal		100% removal	
		D-C1	D-C2	D-C1	D-C2	D-C1	D-C2	D-C1	D-C2
Carbon stocks	Tanzania	-0.91	-0.96	-2.15	-2.26	-3.88	-4.10	-6.21	-6.60
	Cameroon	-2.37	-2.44	-5.13	-5.34	-8.21	-8.78	-11.86	-13.85
	Congo	-0.55	-0.60	-1.20	-1.26	-2.04	-2.23	-2.80	-3.13
	India	-0.79	-0.84	-2.00	-2.09	-3.52	-3.73	-6.03	-6.41
	Peru	-1.12	-1.12	-2.31	-2.35	-3.78	-3.84	-5.50	-5.51
	Panama	-0.66	-0.66	-1.51	-1.59	-2.55	-2.65	-3.44	-3.41
	Costa Rica	-0.45	-0.47	-0.95	-1.05	-1.52	-1.84	-2.04	-2.55
	Malaysia	-0.21	-0.26	-0.36	-0.55	-0.64	-0.70	-0.97	-0.89
	Australia	-0.12	-0.14	-0.20	-0.25	-0.37	-0.37	-0.58	-0.52
	Indonesia	+0.14	+0.07	+0.39	+0.07	+0.57	+0.23	+0.86	-0.12

Volume	Tanzania	-1.52	-1.55	-3.52	-3.60	-6.18	-6.37	-9.98	-10.02
	Cameroon	-1.59	-1.63	-3.51	-3.66	-5.72	-6.00	-8.21	-9.19
	Congo	-0.61	-0.65	-1.27	-1.35	-2.08	-2.24	-2.97	-3.01
	India	-1.10	-1.14	-2.67	-2.76	-4.60	-4.80	-7.79	-8.19
	Peru	-0.43	-0.45	-0.85	-0.85	-1.40	-1.34	-2.07	-2.06
	Panama	-0.42	-0.44	-0.97	-1.00	-1.60	-1.70	-2.23	-2.03
	Costa Rica	+0.16	+0.13	+0.37	+0.26	+0.53	+0.34	+0.91	+0.38
	Malaysia	+0.25	+0.20	+0.57	+0.45	+0.86	+0.81	+1.08	+1.23
	Australia	+0.21	+0.19	+0.57	+0.51	+0.88	+0.90	+1.22	+1.22
	Indonesia	+0.24	+0.17	+0.56	+0.34	+0.87	+0.61	+1.22	+0.71
Relative abundance of large trees	Tanzania	-11.52	-11.79	-25.12	-25.45	-40.69	-42.17	-58.48	-60.03
	Cameroon	-11.14	-11.31	-22.55	-23.53	-35.92	-37.37	-50.19	-54.94
	Congo	-6.93	-6.88	-13.5	-14.07	-22.02	-22.77	-31.36	-33.14
	India	-4.99	-5.39	-12.05	-11.93	-20.29	-20.95	-33.19	-32.70
	Peru	-4.97	-4.83	-9.10	-9.33	-15.58	-15.56	-22.44	-22.57
	Panama	-4.83	-4.96	-9.32	-9.42	-15.37	-15.04	-21.45	-20.41
	Costa Rica	-0.68	-0.76	-1.23	-1.65	-1.72	-2.66	-1.62	-4.43
	Malaysia	+3.87	+3.48	+8.82	+8.17	+13.79	+13.39	+17.97	+19.83
	Australia	+0.94	+0.66	+3.30	+2.81	+5.20	+5.36	+7.30	+6.53
	Indonesia	+2.20	+1.87	+4.70	+3.17	+7.05	+5.52	+10.39	+6.10
Wood density	Tanzania	+0.64	+0.64	+1.51	+1.45	+2.62	+2.50	+4.17	+4.07
	Cameroon	-0.43	-0.51	-0.99	-1.05	-1.72	-1.86	-2.53	-2.93
	Congo	+0.15	+0.14	+0.30	+0.25	+0.43	+0.37	+0.74	+0.57
	India	+0.07	+0.05	+0.05	+0.03	+0.05	+0.01	+0.21	-0.64
	Peru	-0.59	-0.56	-1.29	-1.30	-2.02	-1.95	-2.90	-2.85
	Panama	-0.26	-0.25	-0.58	-0.56	-0.91	-0.89	-1.35	-1.44
	Costa Rica	-0.50	-0.48	-1.04	-1.01	-1.68	-1.63	-2.36	-2.34
	Malaysia	-0.37	-0.37	-0.92	-0.93	-1.43	-1.41	-2.03	-2.01
	Australia	-0.32	-0.32	-0.73	-0.73	-1.22	-1.18	-1.73	-1.64
	Indonesia	-0.18	-0.17	-0.33	-0.30	-0.52	-0.45	-0.68	-0.68

Supplementary Table 2: The percentage of simulation runs in which carbon stocks reduced following simulated extirpations of large-seeded animal-dispersed trees at each site, along with corresponding values for the two control scenarios

**Site**	**% of simulation runs in which carbon stocks were reduced**											
	**Defaunation (Large-seeded species removed)**				**Control-1 (Equal numbers of individuals removed at random)**				**Control-2 (Equal numbers of species removed at random)**			
	**25%**	**50%**	**75%**	**100%**	**25%**	**50%**	**75%**	**100%**	**25%**	**50%**	**75%**	**100%**
Tanzania	92	99	100	100	53	51	52	51	51	33	38	44
Cameroon	95	99	100	100	52	51	52	53	42	38	36	39
Congo	79	90	96	99	52	51	51	53	48	46	45	48
India	83	93	99	100	52	52	52	51	63	37	37	46

Peru	91	97	99	100	50	51	52	51	49	50	50	50
Panama	79	87	94	100	50	51	52	51	54	48	48	51
Costa Rica	78	87	94	100	50	50	50	51	43	41	39	45
Malaysia	70	74	76	79	55	56	52	49	53	47	50	52
Australia	61	66	71	80	52	52	52	49	49	51	52	51
Indonesia	37	23	15	10	51	54	50	51	39	38	40	40

Supplementary Table 3: Output of linear mixed effects model comparing species' maximum diameters, maximum attainable heights and wood densities across seed size and dispersal groups (fixed effects) with site included as a grouping term in the random part of the models.

**Seed dispersal category**	**Maximum diameter (cm) Mean (±1SE)**	**Maximum attainable height (m) Mean (±1SE)**	**Wood density ($g.cm^{-3}$) Mean (±1SE)**
Small-seeded animal-dispersed	35.25 (±1.40)	22.51 (±0.62)	0.60 (±0.01)
Large-seeded animal - dispersed	43.61 (±2.98)	28.45 (±1.22)	0.63 (±0.02)
Abiotically-dispersed	50.92 (±1.78)	32.19 (±0.81)	0.59 (±0.01)

Supplementary Table 4: Dataset and site details for the ten tree community plot data used for simulations

Sl. No.	Country	Location	Dataset identifier	Sampling year	Latitude	Longitude	No. of plots	Plot size (ha)	Plot IDs	Mean annual precipitation (mm)
1	Australia	Mount Lewis, Agapetes, Whyanbeel, Oliver Creek and Fantail	Ecological Archives E095-209 http://dx.doi.org/10.1890/14-0458R.1	2012/ 2013	-16.3	145.2	6	0.5	ep18, ep30, ep35, ep40,ep41,ep44	2600
2	Cameroon	Korup National Park	TEAM-DataPackage-20140701065026_1902	2013	5.06	8.85	6	1	VG-KRP-1 to VG-KRP-6	5300
3	Panama	Barro Colorado Nature Monument - Soberania National Park	TEAM-DataPackage-20140701042113_1315	2011	9.156	-79.78	6	1	VG-BCI-1 to VG-BCI-6	2900
4	Indonesia	Bukit Barisan	TEAM-DataPackage-20140701043656_1496	2012	-5.65	104.44	6	1	VG-BBS-1 to VG-BBS-6	2950
5	Malaysia	Pasoh Forest Reserve	TEAM-DataPackage-20140701070537_1695	2013	3.03	102.13	6	1	VG-PSH-1 to VG-PSH-6	2050
6	Costa Rica	Volcán Barva	TEAM-DataPackage-20140701071921_1728	2013	10.29	-84.06	5	1	VG-VB-1 to VG-VB-4 and VG-VB-9	4000
7	Congo	Nouabalé Ndoki	TEAM-DataPackage-20140701070319_1368	2013	2.5	16.49	6	1	VG-NNN-1 to VG-NNN-6	1670
8	Tanzania	Udzungwa Mountains National Park	TEAM-DataPackage-20140701071705_1335	2013	-7.77	36.87	6	1	VG-UDZ-1 to VG-UDZ-6	2000
9	Peru	Cocha Cashu - Manu National Park	TEAM-DataPackage-20140701045947_1389	2013	-11.91	-71.36	6	1	VG-COU-1 to VG-COU-6	2100
10	India	Central Western Ghats	Ecological Archives E091-216-D1	1997	13.63	75.02	5	1	BSP69, BSP73, BSP74, BS75, BSP104	6750

Supplementary References

1. Adekunle, V. A. J. Conservation of tree species diversity in tropical rainforest ecosystem of south-west Nigeria. *J. Trop. For. Sci.* **18,** 91 (2006).
2. Asase, A., Asiatokor, B. K. & Ofori-Frimpong, K. Effects of selective logging on tree diversity and some soil characteristics in a tropical forest in southwest Ghana. *J. For. Res.* **25,** 171–176 (2014).
3. Bhuyan, P., Khan, M. L. & Tripathi, R. S. Tree diversity and population structure in undisturbed and human-impacted stands of tropical wet evergreen forest in Arunachal Pradesh, Eastern Himalayas, India. *Biodivers. Conserv.* **12,** 1753–1773 (2003).
4. Bradford, M. G., Murphy, H. T., Ford, A. J., Hogan, D. L. & Metcalfe, D. J. Long-term stem inventory data from tropical rain forest plots in Australia: Ecological Archives E095-209. *Ecology* **95,** 2362–000 (2014).
5. Brewer, S. W. & Webb, M. A. H. A seasonal evergreen forest in Belize: unusually high tree species richness for northern Central America. *Bot. J. Linn. Soc.* **138,** 275–296 (2002).
6. Budke, J. C., Jarenkow, J. A. & de Oliveira-Filho, A. T. Tree community features of two stands of riverine forest under different flooding regimes in Southern Brazil. *Flora-Morphology, Distrib. Funct. Ecol. Plants* **203,** 162–174 (2008).
7. Cattanio, J. H., Anderson, A. B. & Carvalho, M. S. Floristic composition and topographic variation in a tidal floodplain forest in the Amazon Estuary. *Rev. Bras. Botânica* **25,** 419–430 (2002).
8. Condit, R. Cocoli 4 ha plot tables. *Smithsonian Tropical Research Institute* (2015). at <http://www.ctfs.si.edu/site/Cocoli>
9. Ewango, C. E. N., Makana, J.-R., Hart, T. & Hart, J. Edoro species abundance tables. *Smithsonian Tropical Research Institute* (2015). at <http://www.ctfs.si.edu/site/Ituri>
10. Bunyavejchewin, S. Huai Kha Khaeng species abundance tables. *Smithsonian Tropical Research Institute* (2015). at <http://www.ctfs.si.edu/site/Huai+Kha+Khaeng>
11. Alvarez, M. La Planada species abundance tables. *Smithsonian Tropical Research Institute* (2015). at <http://www.ctfs.si.edu/site/La+Planada>
12. Tan, S., Itoh, A., Davies, S. & Yamakura, T. Lambir species abundance tables. *Smithsonian Tropical Research Institute* (2015). at <http://www.ctfs.si.edu/site/Lambir>
13. Ewango, C. E. N., Makana, J.-R., Hart, T. & Hart, J. Lenda species abundance tables. *Smithsonian Tropical Research Institute* (2015). at <http://www.ctfs.si.edu/site/Ituri>
14. Zimmerman, J., Thompson, J. & Brokaw, N. Luquillo species abundance tables. *Smithsonian Tropical Research Institute* (2015). at <http://www.ctfs.si.edu/site/Luquillo>
15. Brockelman, W. Y. & Nathalang, A. Mo Singto species abundance tables. *Smithsonian Tropical Research Institute* (2015). at <http://www.ctfs.si.edu/site/Mo+Singto>
16. Condit, R. Sherman species abundance tables. *Smithsonian Tropical Research Institute* (2015). at <http://www.ctfs.si.edu/site/Sherman>
17. Valencia, R. Yasuni species abundance tables. *Smithsonian Tropical Research Institute* (2015). at <http://www.ctfs.si.edu/site/Yasuni>
18. Eggeling, W. J. Observations on the ecology of the Budongo rain forest, Uganda. *J. Ecol.* **34,** 20–87 (1947).
19. Eilu, G. & Obua, J. Tree condition and natural regeneration in disturbed sites of Bwindi Impenetrable Forest National Park, southwestern Uganda. *Trop. Ecol.* **46,** 99–

112 (2005).
20. Fashing, P. J., Forrestel, A., Scully, C. & Cords, M. Long-term tree population dynamics and their implications for the conservation of the Kakamega Forest, Kenya. *Biodivers. Conserv.* **13,** 753–771 (2004).
21. Hall, J., Harris, D., Medjibe, V. & Ashton, M. The effects of selective logging on forest structure and tree species composition in a Central African forest: implications for management of conservation areas. *For. Ecol. Manage.* **183,** 249–264 (2003).
22. Hardy, O. J. & Sonké, B. Spatial pattern analysis of tree species distribution in a tropical rain forest of Cameroon: assessing the role of limited dispersal and niche differentiation. *For. Ecol. Manage.* **197,** 191–202 (2004).
23. Hashimoto, C., Furuichi, T., Tashiro, Y. & Kimura, D. Vegetation of the Kalinzu Forest, Uganda: Ordination of forest types using Principal Component Analysis. *African Study Monographs* **20,** 229–239 (1999).
24. Hernández-Ruedas, M. A. *et al.* Conserving tropical tree diversity and forest structure: the value of small rainforest patches in moderately-managed landscapes. *PLOS One* DOI: 10.1371/journal.pone.0098931 (2014).
25. Htun, N. Z., Mizoue, N. & Yoshida, S. Tree species composition and Diversity at different levels of disturbance in Popa Mountain Park, Myanmar. *Biotropica* **43,** 597–603 (2011).
26. Ipor, I., Sani, H. & Tawan, C. Floristic composition of forest formation at Mahua, Crocker range national park, Sabah. *ASEAN Rev. Biodivers. Environ. Conserv. (ARBEC), July-September* 1–8 (2002).
27. Lopes, A. V., Girão, L. C., Santos, B. A., Peres, C. A. & Tabarelli, M. Long-term erosion of tree reproductive trait diversity in edge-dominated Atlantic forest fragments. *Biol. Conserv.* **142,** 1154–1165 (2009).
28. Lwanga, J. S., Butynski, T. M. & Struhsaker, T. T. Tree population dynamics in Kibale National park, Uganda 1975–1998. *Afr. J. Ecol.* **38,** 238–247 (2000).
29. McLennan, M. R. & Plumptre, A. J. Protected apes, unprotected forest: composition, structure and diversity of riverine forest fragments and their conservation value in Uganda. *Trop. Conserv. Sci.* **5,** 79-103 (2012).
30. Muthuramkumar, S. *et al.* Plant community structure in tropical rain forest fragments of the Western Ghats, India. *Biotropica* **38,** 143–160 (2006).
31. Nath, P. C., Arunachalam, A., Khan, M. L., Arunachalam, K. & Barbhuiya, A. R. Vegetation analysis and tree population structure of tropical wet evergreen forests in and around Namdapha National Park, northeast India. *Biodivers. Conserv.* **14,** 2109–2135 (2005).
32. Nebel, G. *et al.* Structure and floristic composition of flood plain forests in the Peruvian Amazon: I. Overstorey. *For. Ecol. Manage.* **150,** 27–57 (2001).
33. Newbery, D. M., Campbell, E. J. F., Proctor, J. & Still, M. J. Primary lowland dipterocarp forest at Danum Valley, Sabah, Malaysia. Species composition and patterns in the understorey. *Vegetatio* **122,** 193–220 (1996).
34. Okali, D. U. U. & Ola-Adams, B. A. Tree population changes in treated rain forest at Omo Forest Reserve, south-western Nigeria. *J. Trop. Ecol.* **3,** 291–313 (1987).
35. Osuri, A. M., Kumar, V. S. & Sankaran, M. Altered stand structure and tree allometry reduce carbon storage in evergreen forest fragments in India's Western Ghats. *For. Ecol. Manage.* **329,** 375–383 (2014).
36. Parthasarathy, N. Tree diversity and distribution in undisturbed and human-impacted sites of tropical wet evergreen forest in southern Western Ghats, India. *Biodivers. Conserv.* **8,** 1365–1381 (1999).
37. Rajkumar, M. & Parthasarathy, N. Tree diversity and structure of Andaman giant

evergreen forests, India. *Taiwania* **53,** 356–368 (2008).
38. Sanchez, M., Pedroni, F., Eisenlohr, P. V. & Ary T. Oliveira-Filhob. Changes in tree community composition and structure of Atlantic rain forest on a slope of the Serra do Mar range, southeastern Brazil, from near sea level to 1000m of altitude. *Flora-Morphology, Distrib. Funct. Ecol. Plants* **208,** 184–196 (2013).
39. Simbolon, H., Suzuki, E. & Susanti, R. in *The Biodiversity Observation Network in the Asia-Pacific Region* 71–92 (Springer, 2012).
40. Small, A., Martin, T. G., Kitching, R. L. & Wong, K. M. Contribution of tree species to the biodiversity of a 1ha Old World rainforest in Brunei, Borneo. *Biodivers. Conserv.* **13,** 2067–2088 (2004).
41. Stropp, J., Sleen, P. Van der, Assunção, P. A., Silva, A. L. da & Steege, H. Ter. Tree communities of white-sand and terra-firme forests of the upper Rio Negro. *Acta Amaz.* **41,** 521–544 (2011).
42. Tajeukem, V. C., Fongnzossie Fedoung, E., Kemeuze, V. A. & Nkongmeneck, B.-A. Vegetation structure and species composition at the northern periphery of the Boumba-Bek NationAl Park, Southeastern Cameroon. *Afr. Study Monogr.* **49,** 13–46 (2014).
43. Van Do, T., Osawa, A. & Thang, N. T. Recovery process of a mountain forest after shifting cultivation in Northwestern Vietnam. *For. Ecol. Manage.* **259,** 1650–1659 (2010).
44. Velho, N. & Krishnadas, M. Post-logging recovery of animal-dispersed trees in a tropical forest site in north-east India. *Trop. Conserv. Sci.* **4,** 405–419 (2011).
45. Cooper, W. & T Cooper, W. *Fruits of the Australian tropical rainforest*. (CSIRO, 2004).
46. Bendezú, Y. F. *Árboles nativos de la Región Ucayali Peru*. (Pucallpa, Enero, 2014).
47. Dennis, A. J., Schupp, E. W., Green, R. J. & Wescott, D. A. *Seed dispersal: theory and its application in a changing world*. (CABI, 2007).
48. Standley, P. C. *Flora of Costa Rica*. (Field Museum of Natural History).
49. Burger, W. C. *Flora Costaricensis*. (Field Museum of Natural history).
50. Standley, P. C. & Steyermark, J. A. *Flora of Guatemala*. (Field Museum of Natural history, 1937).
51. Macbride, J. F., Gentry, A. H., Dillon, M. O. & Jones, S. B. *Flora of Peru*. (Field Museum of Natural History, 1960).
52. van Steenis, C. G. G. J. *Flora Malesiana*. (Noordhoff-Kolff NV, 1950).
53. Soepadmo, E., Saw, L. G. & Wong, K. M. *Tree flora of Sabah and Sarawak*. (Sabah Forestry Department, Forestry Research Institute Malaysia and Sarawak Forestry Department, 1995).
54. *The Gardens' Bulletin, Singapore*. (Government of Singapore).
55. *Annals of the Missouri Botanical Gardens*. (Missouri Botanical Gardens Press).
56. *Journal of the Arnold Arboretum*. (Harvard University).
57. Africa Tree Database. (2012). at <http://www.africatreedatabase.com/>
58. PROTA. Plant resources of tropical Africa. (2014). at <http://www.prota.org/>
59. Botanic Garden Meise. The Digitised Flora of Central Africa. (2013). at <http://www.br.fgov.be/RESEARCH/DATABASES/FOCA/abbreviations.php>
60. CSIRO. Australian tropical rainforest plants. (2010). at <http://www.anbg.gov.au/cpbr/cd-keys/rfk/>
61. Australian Biological Resources Study. Flora of Australia online. (2014). at <http://www.anbg.gov.au/abrs/online-resources/flora/main-query-styles.html>
62. Smithsonian Institution. Flora of Barro Colorado Island (Stanford University Press, 1978). at <http://biogeodb.stri.si.edu/bioinformatics/croat/home>

63. Smithsonian Institution. STRI herbarium website. at <http://biogeodb.stri.si.edu/herbarium/>
64. INBio. Biodiversity of Costa Rica. (2014). at <http://atta2.inbio.ac.cr/neoportal-web>
65. Castro, E. La Selva florula digital. (2013). at <http://guarea.ots.ac.cr/florula4/>
66. Slik, F. Plants of southeast Asia. at <http://www.asianplant.net/>
67. Ramesh, B. R. *et al.* Biodiversity Information and Co-operation in Taxonomy for Interactive Shared Knowledge Base (BIOTIK): Western Ghats. (2007).
68. India Biodiversity Portal. (2014). at <http://indiabiodiversity.org/>
69. Peter C. van Welzen. Euphorbiaceae of Malesia. (2014). at <http://www.nationaalherbarium.nl/Euphorbs/>
70. Conn, B. J. & Damas, K. Q. Guide to trees of Papua New Guinea. (2005). at <http://www.pngplants.org/PNGtrees/>
71. Zanne, A. E. *et al.* Global wood density database. *Dryad. Identifier: http://hdl. handle. net/10255/dryad* (2009).
72. Royal Botanic Gardens Kew. Seed Information Database V7.1. (2014). at <http://data.kew.org/sid/>
73. World Agroforestry Centre. Agroforestree database. (2014). at <http://www.worldagroforestry.org/resources/databases/agroforestree>
74. Fern, K. Useful tropical plants database. (2014). at <http://tropical.theferns.info/>
75. Paine, T. & Alvarez, P. Plants of Manu National Park. (2014). at <http://manuplants.org/>
76. JSTOR. JSTOR global plants. (2015). at <https://plants.jstor.org/>
77. Royal Botanical Gardens, K. The Herbarium Catalogue. (2014). at http://www.kew.org/herbcat
78. Olson, D. M., *et al.* Terrestrial ecoregions of the world: a new map of life on Earth. Bioscience 51: 933-938. (2001)
